# Supplementary material for: Death and invasive mechanical ventilation risk in hospitalized COVID-19 patients treated with anti-SARS-CoV-2 monoclonal antibodies and/or antiviral agents: A systematic review and network meta-analysis protocol
Source: PLoS One. 2022 Jun 17;17(6):e0270196. doi: 10.1371/journal.pone.0270196 (PMC9205473; doi:10.1371/journal.pone.0270196)
Supplement: S2 File — (DOCX) [file pone.0270196.s002.docx]

# Proposed search strategy in PubMed

| **Search date** | **Search terms & their combination** | **No. Of results** | **Electronic link** | **Pre-identified citations retrieved** | |
| --- | --- | --- | --- | --- | --- |
|  |  |  |  | **DOI** | **Citation position on relevancy wise sorting*** |
| 10-Sep-21 | ("COVID-19"[MeSH Terms] NOT "COVID-19 Vaccines"[MeSH Terms]) AND (randomizedcontrolledtrial[Filter]) | 539 | <https://pubmed.ncbi.nlm.nih.gov/?term=%28%22COVID-19%22%5BMesh%5D%29+NOT+%22COVID-19+Vaccines%22%5BMesh%5D&filter=pubt.randomizedcontrolledtrial&size=200&sort=relevance> | 10.1056/NEJMoa2030340 | 3 |
|  |  |  |  | 10.1056/NEJMoa2028836 | 7 |
|  |  |  |  | 10.1056/NEJMoa2031994 | 8 |
|  |  |  |  | 10.1001/jamainternmed.2020.6615 | 30 |
|  | (("SARS-CoV-2"[Title/Abstract] OR "covid 19"[Title/Abstract] OR "covid 19"[Title/Abstract] OR "coronavirus"[Title/Abstract]) NOT "vaccine*"[Title/Abstract]) AND (randomizedcontrolledtrial[Filter]) | 561 | <https://pubmed.ncbi.nlm.nih.gov/?term=%28%28%28%28SARS-CoV-2%5BTitle%2FAbstract%5D%29+OR+%28COVID-19%5BTitle%2FAbstract%5D%29%29+OR+%28%22COVID+19%22%5BTitle%2FAbstract%5D%29%29+OR+%28coronavirus%5BTitle%2FAbstract%5D%29%29+NOT+%28vaccine%2A%5BTitle%2FAbstract%5D%29&filter=pubt.randomizedcontrolledtrial&ac=no&size=200&sort=relevance> | 10.1056/NEJMoa2030340 | 11 |
|  |  |  |  | 10.1056/NEJMoa2028836 | 12 |
|  |  |  |  | 10.1056/NEJMoa2031994 | 19 |
|  |  |  |  | 10.1001/jamainternmed.2020.6615 | 44 |

*This rank might vary on replication as newly indexed studies in PubMed will populate in the search output
